# Terrestrial water load and groundwater fluctuation in the Bengal Basin

**DOI:** 10.1038/s41598-017-04159-w

**Published:** 2017-06-20

**Authors:** W. G. Burgess, M. Shamsudduha, R. G. Taylor, A. Zahid, K. M. Ahmed, A. Mukherjee, D. J. Lapworth, V. F. Bense

**Affiliations:** 1 Department of Earth Sciences, University College London, London, WC1E 6BT UK; 2 Institute for Risk and Disaster Reduction, University College London, London, WC1E 6BT UK; 3 Department of Geography, University College London, London, WC1E 6BT UK; 4 Bangladesh Water Development Board, Dhaka, Bangladesh; 5 Department of Geology, Dhaka University, Dhaka, 1000 Bangladesh; 6 Department of Geology and Geophysics, Indian Institute of Technology Kharagpur, Kharagpur, West Bengal 721302 India; 7 British Geological Survey, Wallingford, Oxfordshire OX10 8BB UK; 8 Department of Environmental Sciences, Wageningen University, Wageningen, The Netherlands

## Abstract

Groundwater-level fluctuations represent hydraulic responses to changes in groundwater storage due to aquifer recharge and drainage as well as to changes in stress that include water mass loading and unloading above the aquifer surface. The latter ‘poroelastic’ response of confined aquifers is a well-established phenomenon which has been demonstrated in diverse hydrogeological environments but is frequently ignored in assessments of groundwater resources. Here we present high-frequency groundwater measurements over a twelve-month period from the tropical, fluvio-deltaic Bengal Aquifer System (BAS), the largest aquifer in south Asia. The groundwater level fluctuations are dominated by the aquifer poroelastic response to changes in terrestrial water loading by processes acting over periods ranging from hours to months; the effects of groundwater flow are subordinate. Our measurements represent the first direct, quantitative identification of loading effects on groundwater levels in the BAS. Our analysis highlights the potential limitations of hydrogeological analyses which ignore loading effects in this environment. We also demonstrate the potential for employing poroelastic responses in the BAS and across other tropical fluvio-deltaic regions as a direct, *in-situ* measure of changes in terrestrial water storage to complement analyses from the Gravity and Climate Experiment (GRACE) mission but at much higher resolution.

## Introduction

Regional monitoring by the Gravity and Climate Recovery Experiment (GRACE) satellite mission^1^ has prompted concerns over the sustainability of deep groundwater pumping^2^, including across mega-delta regions of Asia^3^ where groundwater meets the needs of over 300 million people. The largest and most densely populated floodplain region is the Bengal Basin, which is the focus of intensive water use^4^, subject to substantial hydrological seasonality^5^ and highly vulnerable to climate change^6^. Here, the Rivers Ganges, Brahmaputra and Meghna (GBM) have a combined annual discharge at their confluence of 1350 km^3^, second only to the River Amazon. The GBM floodplains present the second largest seasonal gravity anomaly observed by the GRACE satellites^5^, driven by variations in terrestrial water storage (ΔTWS), the sum of changes in surface water runoff and storage, soil water and groundwater storage. The uppermost few hundred metres of the Holocene-Pleistocene GBM floodplain sediments constitute the Bengal Aquifer System^7^ (BAS), the source of domestic and irrigation water to over 100 million people^8^. ‘Deep groundwater’ in BAS, greater than 150 m below the floodplain surface, supplies Dhaka as well as many provincial and coastal towns and rural water supply schemes^8^ where it is developed as an alternative to shallow, arsenic-contaminated groundwater^9^. Human dependency on groundwater resources, exposure of groundwater to increasing climatic and anthropogenic stresses, and geogenic constraints on water quality are features of the BAS which are common to other major Asian mega-deltas^9^. The intensity of groundwater abstraction from the BAS has prompted concern^7^ over its sustainability, and focussed attention on the variability of seasonal amplitudes and secular trends in TWS, and especially of groundwater as a component of TWS. Controversy over interpretations of GRACE satellite gravity data across the Bengal Basin centres on assessments of secular groundwater depletion^3, 10^ which range from 1 to 4 km^3^/year; the disparity is partly associated with the choice of scaling factor applied to address amplitude damping that occurs during GRACE processing^11^, and partly with the use of global hydrological models which introduce substantial uncertainty^12^. *In situ* observations offer a valuable and necessary constraint on interpretations of changes in TWS^12, 13^, but despite extensive programmes of shallow water-table monitoring across the Bengal Basin^4, 14^, deeper levels of BAS remain largely unmonitored^7^. Management of the deep groundwater resource in BAS, as elsewhere across south Asia, lacks a sound empirical basis.

**Figure 1:**
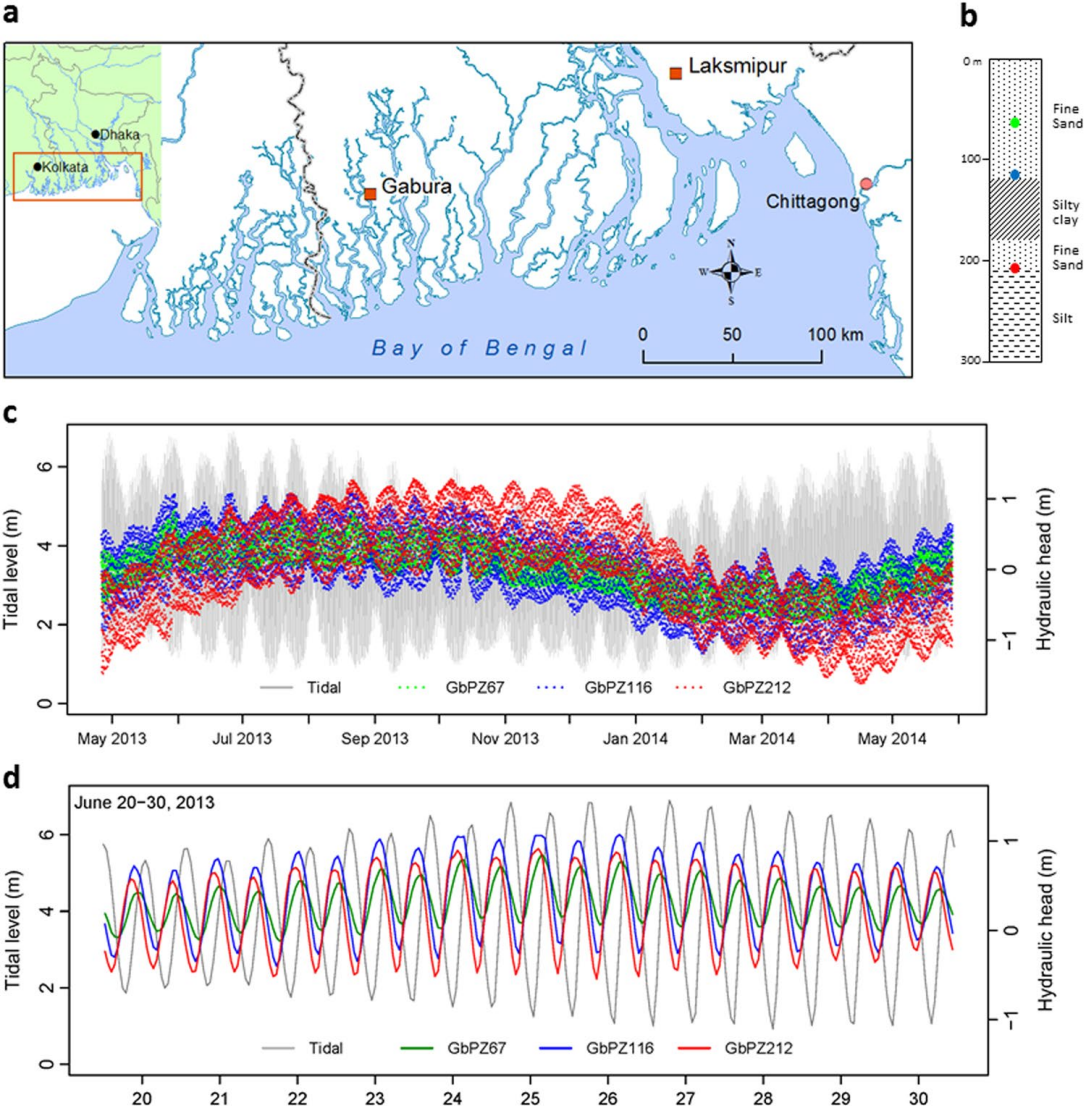
Study sites, hydraulic heads at Gabura and tide level at Chittagong, May 2013 to May 2014. (**a**) Location of the study sites (red squares) in southern Bangladesh (map created using ArcGIS version 10.3.1, https://www.arcgis.com/); (**b**) at Gabura, the open screen measurement points of piezometers GbPZ67 (green, depth 67 m), GbPZ116 (blue, depth 116 m), and GbPZ212 (red, depth 212 m) within the lithological profile; (**c**) the Gabura hydrographs from piezometers GbPZ67, GbPZ116, and GbPZ212 shown as equivalent fresh water head, and Chittagong tide level (grey), relative to the time series averages, May 2013 to May 2014; (**d**) the Gabura piezometer groundwater heads and the Chittagong tide, relative to the time series averages, 20^th^ June to 30^th^ June 2013.

At two sites in southern Bangladesh (Fig. 1, and Supplementary Information) we recorded absolute groundwater pressure and atmospheric pressure at hourly intervals over one year in three closely-spaced boreholes screened at depths between 65 m and 250 m. For construction and completion details of the boreholes see Methods. At Gabura the boreholes GbPZ67, GbPZ116 and GbPZ212 (the numbers indicate the depth to the top of the screen in metres) are situated a few tens of metres from a tidal channel within the GBM delta; shallow groundwater is saline^8^ and groundwater pumping is insignificant. The Laksmipur boreholes LkPZ91, LkPZ152 and LkPZ244 are situated on the Lower Meghna floodplain, 10 km east of the River Meghna and 8 km from Laksmipur municipal boreholes which pump from 270–300 m depth. From the *in-situ* measurements at both sites we derived an hourly record of equivalent fresh water head at the depth of each borehole screen after correcting raw data for atmospheric pressure and salinity-density effects (see Methods). Environmental head^15^ has been proposed for use when vertical groundwater flux is sought and vertical variations in groundwater density occur. Following a recent critical analysis^16^, we used fresh water head to compare the temporal variation of groundwater head at specified depths in borehole water columns of constant density, where vertical flow components are not sought.

## Results

### Groundwater hydrographs at a coastal site

**Figure 2:**
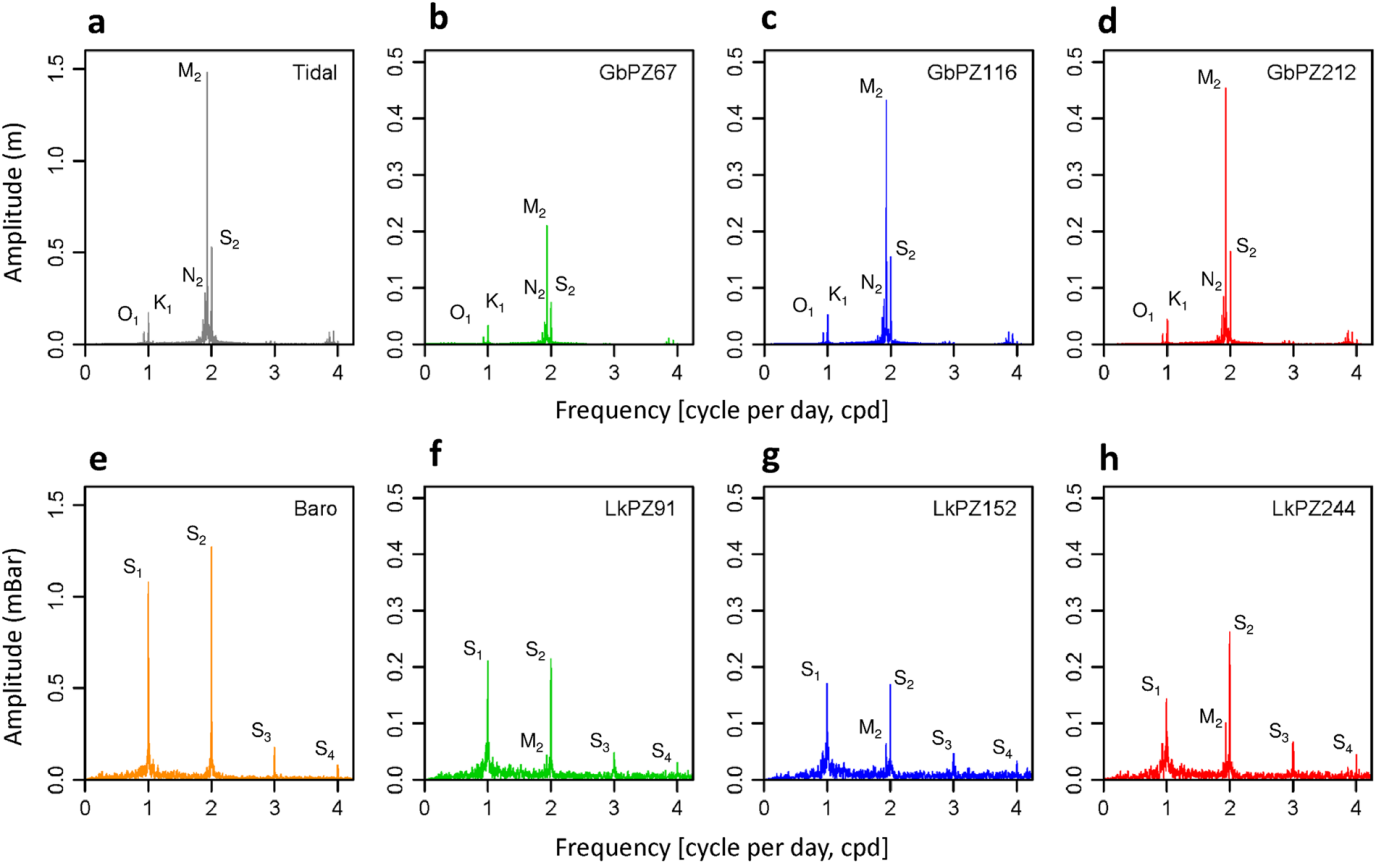
Amplitudes of the tidal and atmospheric signals and groundwater responses at Gabura and Laksmipur. Top: amplitude *versus* frequency for (**a**) the Chittagong tide and (**b**–**d**) the groundwater heads at GbPZ67, GbPZ116 and GbPZ212 respectively, 1^st^ June 2013 to 31^st^ May 2014, showing the principal solar-lunar tidal components^17^ O_1_, K_1_, N_2_, M_2_ and S_2_, and additional minor components (see text). Bottom: amplitude *versus* frequency for (**e**) atmospheric pressure at Laksmipur, and (**f**–**h**) groundwater pressure measured in piezometers LkPZ91, LkPZ152 and LkPZ244 respectively, over the period of hydrograph recession between 1 November 2013 and 30 April 2014.

The Gabura hydrographs (Fig. 1) demonstrate distinctive periodic oscillations containing the principal solar-lunar tidal frequencies^17^ O_1_, K_1_, N_2_, M_2_ and S_2_ and the minor components^18^ MN_4_ and M_4_ (Fig. 2). The oscillations are close to synchronous at all three depths of measurement and amplitudes increase between GbPZ67 and GbPZ116; a summary of amplitudes for specific frequencies is given in Table 1. Subsurface hydraulic responses to a surface sinusoidal hydraulic impulse would dissipate exponentially with depth^19^ according to *T*, the period of the impulse, and *D*, the vertical hydraulic diffusivity (vertical hydraulic conductivity, *K*_*v*_, divided by specific storage, *S*_*s*_) of the sediments. For the BAS, a value of 1.5 × 10^−4^ m^2^/s for *D* derived from *K*_*v*_ and *S*_*s*_ determined by inverse modelling^20^, suggests that hydraulic penetration of the diurnal tidal signal would be less than 10 m; the spring-neap tidal signal should not penetrate beyond a few tens of metres. Therefore we interpret the undamped periodic oscillations at up to 212 m depth as the fluid pressure changes in this coastal, confined and unconsolidated aquifer due to mechanical loading and unloading of the aquifer by tidal water movements above the aquifer surface, commensurate with analytical treatments^21–23^.

**Table 1:** Summary of the frequency components and amplitudes of the tidal and atmospheric signals and the groundwater responses at the Gabura and Laksmipur monitoring boreholes.

Notation and source of tidal/atmospheric frequency components		O_1_	S_1_	K_1_	N_2_	M_2_	S_2_	S_3_	MN_4_	M_4_	S_4_
		lunar diurnal	solar diurnal	lunar-solar diurnal	lunar elliptic semi-diurnal	lunar semi-diurnal	solar semi-diurnal	solar ter-diurnal	shallow water quarter-diurnal	shallow water overtides of principal lunar	solar quarter-diurnal
Frequency (cpd)		0.930	1.000	1.003	1.896	1.933	2.000	3.000	3.828	3.864	4.000
Tidal spectrum amplitude (m)	Chittagong tide	0.067	—	0.171	0.279	1.482	0.532	—	0.028	0.065	—
	GbPZ67	0.013	—	0.033	0.039	0.210	0.074	—	0.004	0.010	—
	GbPZ116	0.020	—	0.052	0.080	0.432	0.155	—	0.010	0.022	—
	GbPZ212	0.018	—	0.045	0.084	0.454	0.164	—	0.012	0.024	—
Atmospheric spectrum amplitude (mBar)	Atmospheric pressure	—	1.076	—	—	—	1.269	0.175	—	—	0.079
	LkPZ91	—	0.210	—	—	0.044	0.214	0.048	—	—	0.031
	LkPZ152	—	0.170	—	—	0.064	0.169	0.046	—	—	0.033
	LkPZ244	—	0.144	—	—	0.101	0.262	0.067	—	—	0.044

Frequency component names follow the U.S. National Oceanic and Atmospheric Administration Tide and Current Glossary^18^. Note that the M_2_ lunar semi-diurnal component in the Laksmipur (Lk) measurements is a response to earth tides.

### Groundwater hydrographs at an inland site

**Figure 3:**
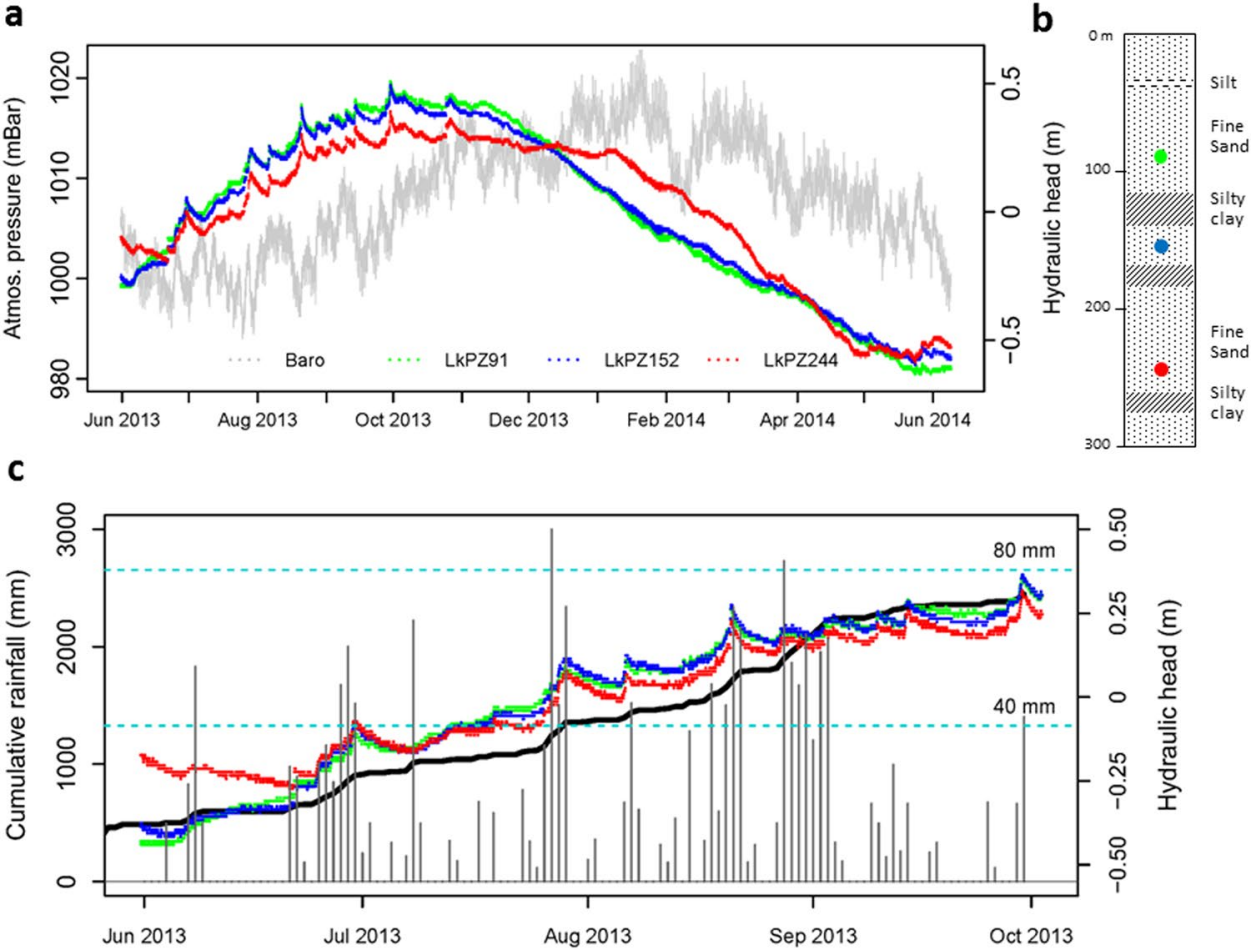
Hydraulic heads and rainfall at Laksmipur, June 2013 to June 2014. (**a**) The Laksmipur hydrographs at piezometers LkPZ91 (green, depth 91 m), LkPZ152 (blue, depth 152 m), LkPZ244 (red, depth 244 m) shown as equivalent fresh water head relative to the time series averages, and contemporaneous atmospheric pressure measured at the site; (**b**) the open screen measurement points of piezometers LkPZ91 (green), LkPZ152 (blue), and LkPZ244 (red) within the lithological profile; (**c**) daily rainfall (vertical bars, scale given as horizontal blue dashed lines) and cumulative rainfall (black line) superimposed on the hydraulic heads of LkPZ91, LkPZ152 and LkPZ244 as in (**a**). Note that the daily rainfall measurements are from a rain gauge 7 km distant from the Laksmipur multi-level piezometer site (Supplementary Information).

**Figure 4:**
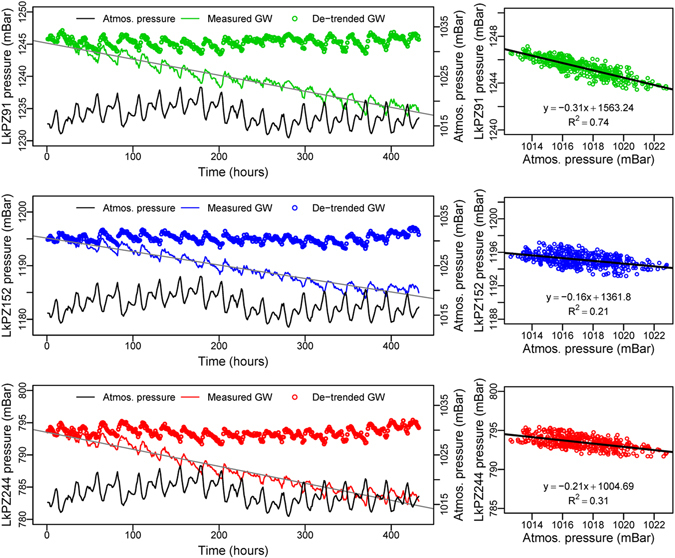
Barometric efficiency, *B*, determined from the inverse relationship between atmospheric pressure and groundwater pressure. Left: Atmospheric pressure (black line) and measured groundwater (GW) pressure at LkPZ91 (green line), LkPZ152 (blue line) and LkPZ244 (red line) with the linear recession trends (grey lines) and as de-trended groundwater pressure series (open circles, colours as above); time is in hours, from 00.00 hours on 14/1/2014 to 24.00 hours on 31/1/2014. Right: De-trended groundwater pressure at LkPZ91 (green), LkPZ152 (blue) and LkPZ244 (red) versus atmospheric pressure, giving the barometric efficiency, B, as the negative value of the linear regression slope.

At Laksmipur (Fig. 3), tidal water loading influences are absent; the hydrographs are characterised by a sequence of episodic increments superimposed on a rising trend throughout the monsoon season. Periodic components are minor compared to Gabura and are dominated by responses to the atmospheric frequency signals S_1_ and S_2_ (Fig. 2), of which only the diurnal component S_1_ shows a consistent trend with depth. During undisturbed periods of dry-season recession, a clear inverse relationship between atmospheric pressure and groundwater pressure is revealed (Fig. 4). As at Gabura, groundwater head variations at Laksmipur are close to simultaneous, within a 2–3 hour time period, and of similar magnitude at the three depths of measurement. Whereas a surface hydraulic impulse undergoes delay and dissipation with depth dependent on the sediment hydraulic diffusivity^24^, neither are evident in the Laksmipur hydrographs. Therefore the episodic increments cannot be explained as the hydraulic consequence of groundwater storage replenishment. Nevertheless the increments are closely associated in time with periods of heavy rainfall (Fig. 3c), measured at a gauge 7 km distant from the piezometer site (Supplementary Information). We interpret the increments in groundwater head at LkPZ91, LkPZ152 and LkPZ244, by analogy with the tidal loading situation at Gabura, as the almost instantaneous hydraulic consequence of the aquifer poroelastic response to terrestrial water loading associated with episodic monsoonal saturation and flooding of the land surface. The rising groundwater heads also track cumulative rainfall (Fig. 3c), which acts as a proxy for surface water loading. Therefore we interpret the coherent seasonal trend recorded between 91 and 244 m depth at Laksmipur as the fluid pressure response to mechanical loading above the aquifer surface through accumulation of terrestrial water as the monsoon progresses. By inverse analogy with the Laksmipur hydrograph rising limbs, we interpret the extended groundwater head recessions principally as the response to unloading of the land surface through a combination of river flood recession, surface water drainage, shallow groundwater drainage and evapotranspiration. The influence of deep groundwater flow may be present in the delay to the start of recession at LkPZ244, the deepest Laksmipur piezometer, relative to LkPZ91 and LkPZ152 (Fig. 3a), but in general it is subordinate to the hydro-mechanical response over the time period of the data record. We note the similarity in the magnitude of annual fluctuation in head (of order 1 m) at LkPZ244 with that at GbPZ212, the deepest point of measurement at Gabura, and suggest this is due to a similar monsoonal water loading experienced at the two locations.

## Discussion

### Poroelastic effects in the Bengal Aquifer System

At both the coastal and the inland sites, over the annual duration of measurements, the data indicate groundwater heads responding dominantly to mechanical loading and unloading due to changes in terrestrial water storage above the aquifer surface by tidal and hydrological processes acting over periods ranging from hours to months and determined by poroelasticity; the effects of groundwater flow are subordinate. Poroelastic responses in the BAS have indeed been predicted^25^. They are favoured by the low topographic relief which restricts gravitational groundwater flow^26^, the low vertical hydraulic conductivity which further constrains depth of groundwater circulation^20, 27^, and the pronounced sediment compressibility^5^ reflected in high *S*_*s*_. Heavy monsoon rainfall and deep surface water flooding^10^ provide a large water load. Hydraulic diffusivity, *D*, controls the relative importance of poroelastic effects as compared to transient groundwater flow on the hydraulic heads of an aquifer. The vertical hydraulic diffusivity of BAS is low because the many silt-clay layers distributed throughout the fluvio-deltaic sediments^20, 27^ impose a low value of *K*_*v*_, and the sediment compressibility, hence S_s_, is high^5^.

We focus on the short-term groundwater pressure transients up to annual time periods because these are the transients that are definitive of the poroelastic response. Projection of secular trends requires a multi-year record, therefore no assessment of potentially longer-term trends linked to groundwater abstraction has been made. The hydrographs have likely not captured the start of the 2013 monsoon season, so any assessment of longer-term trends might be highly misleading. From the strong groundwater pressure responses to mechanical loading, associated poroelastic deformation of the aquifer would be expected, the nature and scale of deformation depending on the drainage condition^28^. No actual measurements of aquifer deformation are available for BAS, although a clear seasonal deflection of the ground surface across Bangladesh with an amplitude of approximately 0.06 m has been observed and associated with larger-scale, deep crustal deformation by monsoon water loading across the entire Bengal Basin^5^.

Fourier analysis^17, 29^ of the time series of tidal and atmospheric signals and groundwater heads provides detail of the periodic loads on the BAS (Fig. 2, Table 1). At Gabura, groundwater responses to the solar-lunar tidal components are undamped and in-phase to the depth of GbPZ212, critical evidence for the dominantly mechanical response of the aquifer at these depths to periodic tidal loading at the surface. At Laksmipur, groundwater responses to the diurnal and semi-diurnal atmospheric frequencies are likewise largely undamped to the depth of LkPZ244; they enable a direct evaluation of barometric efficiency from the relative amplitudes of the atmospheric signals and their groundwater responses. At Laksmipur the main lunar semi-diurnal (M_2_) component of the earth tide is also evident, at an increasing amplitude with depth, while at Gabura any earth tide response is masked by the dominant tidal load responses at the frequencies common^17^ to all ocean and earth tides.

### Implications for groundwater resources

Under the conditions found at Gabura and Laksmipur, the conventional principle of groundwater resource monitoring, which assumes negligible loading influence, is invalid. Borehole water levels do not fluctuate solely, or principally, on account of changes in groundwater storage: rising water levels in deep monitoring boreholes do not necessarily indicate replenishment and falling water levels may not imply depletion of groundwater storage. On the contrary, groundwater heads at over 200 m depth at one coastal location and one inland location (Gabura and Laksmipur respectively) respond rapidly, within a few hours, to distinctive loading signals, consistent with poroelastic^28^ deformation being the dominant process controlling the observed changes in hydraulic head. We have made similar observations^30^ at six additional sites between West Bengal, India, and eastern Bangladesh; the phenomenon may therefore be widespread throughout the Bengal Basin. Additional observations are necessary to confirm the scale of the poroelastic responses in the variety of geomorphological and hydrological contexts of the Basin, but it is likely that assessments of groundwater recharge^31^ and groundwater storage changes^10, 32^ across the Basin, based solely on hydraulic analyses of groundwater hydrographs, need to be revisited. Uncoupled, solely hydraulic treatments of transient groundwater conditions which ignore the possibility of loading effects, including the calibration of transient groundwater flow models^33^ using groundwater hydrographs, must likewise be in doubt.

### Geological weighing lysimetry in the Bengal Basin

Observations of groundwater levels responding to changes in near-surface water mass, equivalent to those reported here, have been made in diverse geological and hydrological environments worldwide and underlie the concept of ‘geological weighing lysimeters’^34–39^. The approach employs the equation governing one-dimensional, transient groundwater pressure $(\frac{\partial p}{\partial t})$ in permeable, elastically compressible sediments under purely vertical strain^35^:

1 $$\frac{\partial p}{\partial t}=D\frac{{\partial }^{2}p}{\partial {z}^{2}}+C\frac{\partial {\sigma }_{T}}{\partial t}$$

where the first term on the right describes flow-induced changes, and the second term describes the effect of changes in vertical stress, or mass loading. *D* is the sediment hydraulic diffusivity (hydraulic conductivity divided by specific storage); *C* is the sediment loading efficiency (dimensionless), which has a value between 0 and 1 according to the distribution of surface load between the confined water and the solid matrix; and *σ*_*T*_ is the total vertical stress applied as a mechanical load on the formation. Conventional hydrogeological analysis neglects the second term on the right of Eq. (1), effectively uncoupling the flow field from the stress field on the premise that poroelastic effects are normally secondary to hydraulic effects. Where groundwater flow is negligible, however, groundwater pressure responds instantaneously to addition or subtraction of mechanical load^19^, as governed by:

2 $$\frac{\partial p}{\partial t}=C\frac{\partial {\sigma }_{T}}{\partial t}$$

Groundwater head response to tidal loading at Gabura potentially provides an empirical means for a direct measure of loading efficiency *C*, the proportional change in groundwater pressure relative to change in total vertical stress, and hence the possibility of applying geological weighing lysimetry in the Bengal Basin. However the actual tidal signal at Gabura, where the coastline is densely dissected by tidal channels, is unknown and likely considerably different to the Chittagong tide. Therefore we determined *C* indirectly through its relationship^21^ with barometric efficiency, *B*, where:

3 $$B+C=1$$

Determination of *B* requires that influences on groundwater head other than atmospheric pressure can be estimated and subtracted from the measurements, or are minor in comparison to the atmospheric effects, or are absent^17^. At Gabura, barometric effects are obscured by the tidal responses which are more than two orders of magnitude greater than the atmospheric signal (Fig. 2). However at Laksmipur, during undisturbed periods of dry-season recession (Fig. 3), the trend of groundwater head decline can be determined and subtracted to reveal the inverse relationship between atmospheric pressure and groundwater pressure (Fig. 4). Barometric efficiency determined by linear regression of this relationship^17^ is 0.31 ± 0.02 (LkPZ91), 0.16 ± 0.03 (LkPZ152) and 0.21 ± 0.03 (LkPZ244), giving values for loading efficiency, *C*, from Eq. (3) as 0.69 ± 0.02 (LkPZ91), 0.84 ± 0.03 (LkPZ152) and 0.79 ± 0.03 (LkPZ244); ranges are ± twice the standard error in each case (Table 2). The mean value of *C* by this method is 0.77. The Fourier method^17, 29^ applied to the solar diurnal, S_1_, and semi-diurnal, S_2_, atmospheric frequency components and groundwater responses (Fig. 2, Table 1) indicates values for *B* of 0.19-0.16 (LkPZ91), 0.16-0.13 (LkPZ152) and 0.13-0.21 (LkPZ244). The mean values for loading efficiency, *C*, from Eq. (3) by the Fourier method are 0.82 (LkPZ91), 0.85 (LkPZ152) and 0.83 (LkPZ244). The two approaches illustrate the variability in *C* for BAS and yield values consistent with measurements of loading efficiency at geological weighing lysimeter sites elsewhere^34, 36, 37^

**Table 2:** Monsoon seasonal TWS accumulation over the Laksmipur piezometer sensing areas.

	Loading efficiency *C*		Maximum fresh water head (m)	Minimum fresh water head (m)	Accumulated fresh water head (m)	Accumulated head (m), corrected for *B*	Accumulated TWS (m)	
	(regression)	(Fourier, S1–S2)					(*C*_r_)	(*C*_F_)
LkPZ91	0.69 ± 0.02	0.81–0.84	6.78	5.98	0.80	0.80	1.15	0.97
LkPZ152	0.84 ± 0.03	0.84–0.87	7.69	6.91	0.78	0.77	0.92	0.91
LkPZ244	0.79 ± 0.03	0.87–0.79	3.69	3.11	0.58	0.59	0.74	0.71

Ranges of values for loading efficiency, *C*, by regression analysis are ± twice the standard error; ranges of *C* by Fourier analysis are by reference to the S_1_ and S_2_ atmospheric frequency components determined independently. *C*_r_ denotes application of *C* as determined by regression; *C*_F_ denotes application of *C* as determined by Fourier.

but uncertainty remains in the evaluated range between 0.67 and 0.87. There is no consistent trend with depth.

Applying the Laksmipur boreholes as geological weighing lysimeters^34^ (see Methods) we evaluated seasonal inundation over the 2013 monsoon period, ΔTWS_m_ (Table 2). In an infinite, uniform half-space, 90% of the terrestrial water loading signal derives from a ‘sensing area’ of radius approximately ten times the borehole depth^35^, *i*.*e*. 3 km^2^ (LkPZ91), 10 km^2^ (LkPZ152) and 20 km^2^ (LkPZ244). We determined ΔTWS_m_ directly from the accumulation of groundwater head (as equivalent fresh water head) recorded between the start (h_1_) and end (h_2_) of the rising limbs of the groundwater hydrographs and accounting for loading efficiency, *C*, where:

4 $${{\rm{\Delta }}\mathrm{TWS}}_{{\rm{m}}}=({{\rm{h}}}_{2}\mbox{--}{{\rm{h}}}_{1})/C$$

By this analysis, using the estimates of *C* from evaluation of *B* by linear regression and Fourier analysis, seasonal inundation during the 2013 monsoon period averaged 0.90 m effective water depth, ranging from 1.15 m (LkPZ91) to 0.71 m (LkPZ244) and possibly underestimating the full seasonal inundation if, as noted, the hydrographs have not captured the start of the monsoon. As the integrated seasonal accumulation of water mass, ΔTWS_m_ is the same variable as measured by GRACE at a scale^1^ of approximately 10^5^ km^2^, assessments of which range from 0.49 to 0.75 m per monsoon season across the GBM floodplains over the period 2003–2007^5, 10^ and 0.51 m for 2013 (Supplementary Information). Differences potentially arising from the large contrast in observation scale highlight possible within-basin variability of ΔTWS. Discrepancy may additionally be due to amplitude damping that occurs during GRACE processing^11^, for which no ground-truth basis for a scaling correction currently exists.

### Modelling investigation of depth and extent of the loading influences

**Figure 5:**
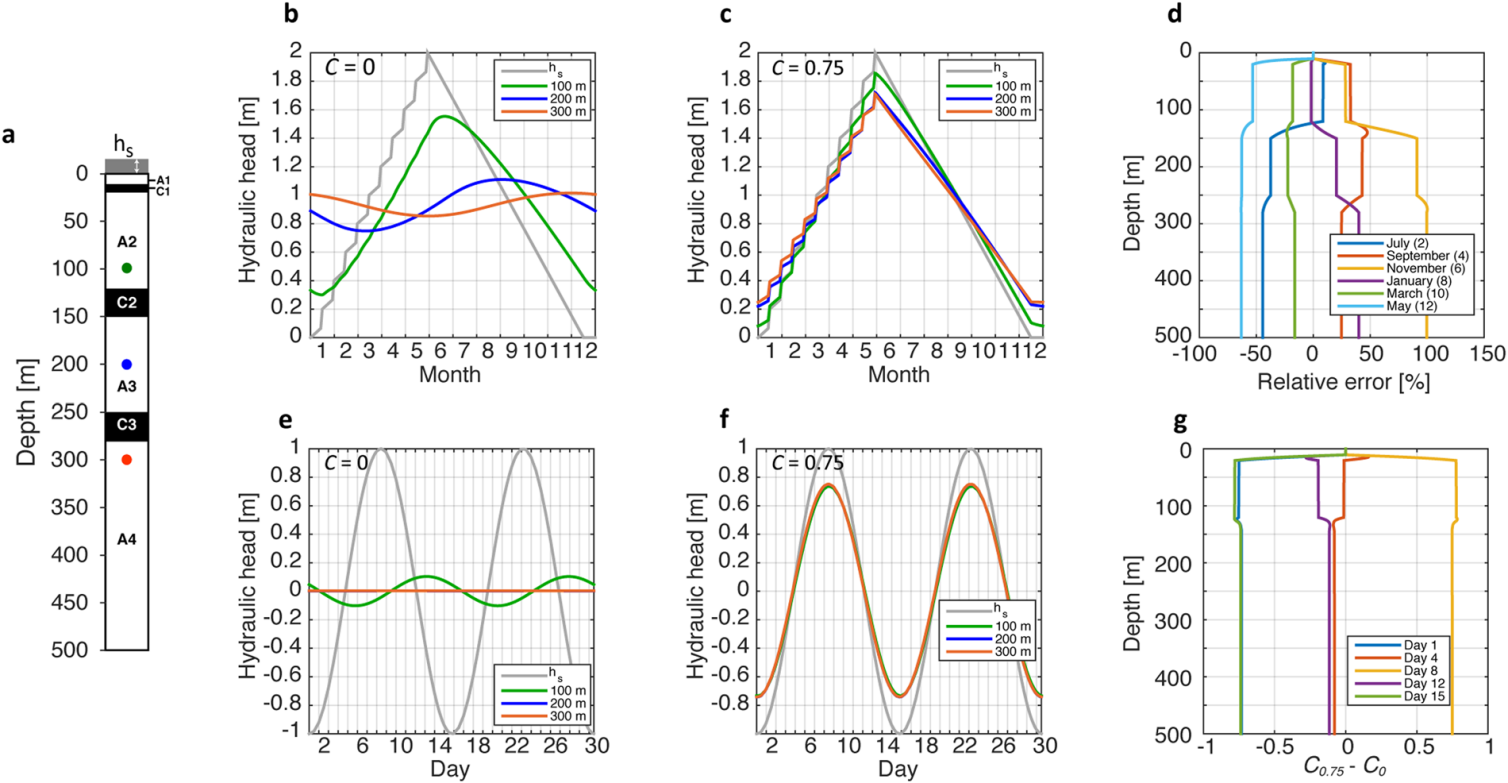
Model simulations showing the influences of poroelasticity. (**a**) Model configuration of permeable (A1-4) and confining (C1-3) units, with parameter values (A1-4) *K* = 10^−5^ m/s, *S*_*s*_ = 10^−5^ m^−1^; (C1-3) *K* = 10^−8^ m/s, *S*_*s*_ = 10^−4^ m^−1^. A column of fresh water at the surface (*h*_*s*_) represents surface water depth. Colour-coded dots at depths 100 m (green), 200 m (blue) and 300 m (red) show where hydraulic head is reported for a monsoonal (**b** and **c**) and a tidal (**e** and **f**) scenario for different values of *C*. A value for *C* = 0.75 was chosen as representative for the BAS, as described in the text. In (**d**) the relative difference between the monsoonal scenario simulations with *C* = 0 and *C* = 0.75 is expressed as relative error calculated as $\frac{{C}_{0.75}-{C}_{0}}{{C}_{0}}$; in (g) the absolute difference between the tidal scenario simulations with *C* = 0 and *C* = 0.75 is illustrated.

To further explore the hydro-mechanical BAS response to ΔTWS, we assembled characteristics of the aquifer in a numerical model of coupled vertical strain and groundwater flow in one-dimension (see Methods). A value of *C* = 0.75 was chosen as representative for the BAS, approximately central to the range of determined values. The significance of uncertainty in *C* was explored by applying the minimum (*C* = 0.67) and maximum (*C* = 0.87) values of the determined range. The simulations reproduce the characteristic features of the Gabura and Laksmipur hydrographs (Fig. 5, and Supplementary Information). Results are consistent with conclusions of hydrograph analysis that the deep groundwater head variations monitored over a twelve month period are inexplicable without reference to poroelasticity. The purely hydraulic response, when *C* = 0 (Fig. 5b and e), is significantly overprinted by poroelastic effects at all values of *C* within the determined range when vertical hydraulic diffusivity values representative of BAS are applied (Fig. 5c and f, and Supplementary Information). The modelled hydrograph peaks are synchronous with the surface water loading signal, and the poroelastic effect becomes increasingly dominant with depth (Fig. 5d and g). In the tidal scenario, the shorter period of the spring/neap tidal signal compared to monsoonal forcing leads to shallower penetration of the hydraulic response, so the poroelastic influence is more uniformly dominant throughout the stratigraphic column. Modelling therefore confirms that ignoring poroelastic effects on hydraulic head would lead to false conclusions in respect of aquifer replenishment and depletion.

The depth at which poroelastic effects become important can vary greatly from one location to another, depending largely on the permeability structure of the overlying formations. Poroelastic dominance has been recorded at 40 m (New Zealand^34^) and 52 m (South Carolina, USA^36^). The model results (Fig. 5 and Supplementary Information) suggest that poroelastic effects in BAS may become significant at depths much shallower than reported here for Gabura and Laksmipur. In the monsoon scenarios with *C* = 0.75, poroelasticity imposes a relative difference exceeding 25% of the purely hydraulic response at depths as shallow as 25 m (Fig. 5d), thus affecting water levels over approximately half the hydrological year including the monsoon season (when groundwater levels are higher than otherwise) and the dry season (when groundwater levels are lower than otherwise). Boreholes used for groundwater level monitoring by the Bangladesh Water Development Board range from less than 5 m to 77 m depth (median 30 m, n = 1035)^4^; 76% (n = 790) are deeper than 25 m and are therefore vulnerable to significant poroelastic disturbance where confined conditions pertain.

## Conclusions

Our findings expose the magnitude of the poroelastic effects of terrestrial water loading on the largest fluvio-deltaic aquifer system in south Asia. They challenge the validity of the traditional philosophy of borehole water level measurement as a means to monitor groundwater storage and recharge in BAS^10, 31, 32^ and possibly other extensive fluvio-deltaic aquifers of the Asian mega-deltas. More generally, they challenge the application of uncoupled, solely hydraulic treatments of transient groundwater conditions in these regions^33^. Strategies for management and monitoring of groundwater resources in the fluvio-deltaic aquifers of south Asia should consider a broader, coupled hydro-mechanical approach which acknowledges poroelasticity.

Applying determinations of loading efficiency at Laksmipur, we have made the first direct, *in-situ* determination of ΔTWS in a tropical fluvio-deltaic environment. Results complement GRACE determinations made across much larger spatial scales and at lower temporal resolution. We propose that multiple nests of deep boreholes across the GBM floodplains could be used to establish the scale of within-basin variability of ΔTWS and to allow a direct calibration^25^ of the magnitude of scaling factors applied in GRACE assessments.

## Methods

### Determination of equivalent fresh water head

We recorded absolute groundwater pressure in three boreholes at each of two sites in southern Bangladesh (Table 3) using ‘*In-Situ* Rugged Troll 100’ piezoresistors suspended at a known depth approximately 10 m below the static water level and set to record at hourly intervals. The boreholes were drilled by direct-circulation rotary drilling, and completed with PVC casing and a 10 m length of screen at the base, the annular space between casing and borehole wall being sealed with a bentonite and clay mixture to ensure isolation of the head measurement interval. We installed a barometric recorder in the air-filled section of one piezometer at each site to record atmospheric pressure. We evaluated groundwater density at each piezometer from electrical conductivity, a proxy for total dissolved solid content, and temperature measured in the discharge line of a submersible pump. Three well volumes were flushed prior to making measurements. We took the ratio of dissolved solids concentration in mg/L to electrical conductivity in μS/cm as 0.65 and determined density as function of temperature and concentration following standard relationships^40^.

**Table 3:** Screen depths and groundwater electrical conductivity, temperature, and density for the boreholes at Gabura and Laksmipur, southern Bangladesh; borehole names include numbers which indicate the screen depth in metres, at which the head determinations apply.

	Gabura			Laksmipur		
	GbPZ67	GbPZ116	GbPZ212	LkPZ91	LkPZ152	LkPZ244
Depth (m, below ground level)	67	116	212	91	152	244
Electrical conductivity (μS/cm)	8150	9410	3270	19460	18300	539
Temperature (°C)	27.4	28.0	29.0	27.2	26.9	28.7
Density (kg/m^3^)	1000	1001	998	1006	1005	996

To determine equivalent fresh water head at each piezometer screen, first we subtracted barometric pressure from *in-situ* measurements of absolute groundwater pressure to obtain gauge pressure, *p*_*w*_. Then we computed equivalent fresh water head, *h*_*f*_ (m, relative to local datum), taking account of the piezoresistor depth and the groundwater density:

5 $${h}_{f}=({\rm{e}}-{{\rm{z}}}_{1})+({p}_{w}+{p}_{t})/({1000}^{\ast }{\rm{g}})$$

6 $${p}_{t}=({{\rm{z}}}_{1}\mbox{--}{{\rm{z}}}_{2}){\rho }_{f}\cdot {\rm{g}}$$

where:

e is elevation of the piezometer reference measurement point relative to local datum (m);

z_1_ is depth of the piezometer open section, below the piezometer reference point (m);

*p*_*w*_ is gauge pressure at the piezoresistor (Pa);

*p*_*t*_ is pressure at the piezometer screen due to fluid below the piezoresistor (Pa);

z_2_ is depth of the piezoresistor, below the piezometer reference point (m);

*ρ*_*f*_ is groundwater density (kg/m^3^);

g is acceleration due to gravity.

### Frequency analysis of the periodic loading signals and groundwater responses

Frequency analysis of the atmospheric, tidal and groundwater time series was implemented using the Fast Fourier Transform (FFT) algorithm in the R programming language^41^ (version 3.0.1). FFT was performed after the hourly time series data were detrended using a 25-point (i.e. 25-hour) moving average in order to remove the lower frequency components.

### Evaluating monsoon season accumulation of terrestrial water storage (TWS_m_)

Monsoon seasonal TWS accumulation was determined (Table 2) for the sensing area of each piezometer at the Laksmipur site from the difference in the equivalent fresh water head calculated at the time of maximum head, at 5.00 am on 30/9/2013 in all cases, and the minimum head at the onset of the monsoon evident at 11.00 am 4/6/2013 (LkPZ91), midday 4/6/2013 (LkPZ152), and midday 20/6/2013 (LkPZ244), using Eq. (4) after correction for barometric effects. Acknowledging uncertainty in the value of loading efficiency, *C*, the range of values obtained from evaluation of barometric efficiency, *B*, by linear regression and Fourier analysis was applied.

### Modelling coupled vertical strain and groundwater flow

We applied a numerical model to simulate the combined poroelastic and hydraulic response of the BAS aquifer using a generalised layered representation of the BAS hydrostratigraphy (Fig. 5) and an equivalent uniform representation (Supplementary Information). We used the generic finite-element code FlexPDE (pdesolutions.com) for this purpose, which has been tested in earlier modelling studies to simulate poroelastic effects^42^. Models were one-dimensional and solve a hydraulic head based version of Eq. (1). Values of vertical hydraulic conductivity, *K*_*v*_, and specific storage, *S*_*s*_, are consistent with the uniform BAS aquifer representation in a basin-scale numerical model^20^. A single value of loading efficiency, *C*, is applied (Fig. 5), making the model results indicative rather than representative of a specific site. The full range of determined values of *C*, including uncertainty, was applied in successive runs of the uniform aquifer representation (Supplementary Information). As a surface boundary condition, the hydraulic head is varied in accordance with an idealized monsoonal or spring/neap tidal loading scenario representing the changing depth of water at the surface. At the base the of the model domain no fluid flow is allowed. Models were run for a time-period of ten years at which point a dynamic steady state is reached and results were analysed.

### Data Availability

The datasets both generated and analysed during the current study are available from the corresponding author on reasonable request.

## Electronic supplementary material

Terrestrial water load and groundwater fluctuation in the Bengal Basin
