# Supplementary material for: Terrestrial water load and groundwater fluctuation in the Bengal Basin
Source: Sci Rep. 2017 Jun 20;7:3872. doi: 10.1038/s41598-017-04159-w (PMC5478667; doi:10.1038/s41598-017-04159-w)
Supplement: Supplementary file 1 — Terrestrial water load and groundwater fluctuation in the Bengal Basin [file 41598_2017_4159_MOESM1_ESM.pdf]

Terrestrial water load and groundwater fluctuation in the Bengal Basin

*[1]Burgess, W.G., [2]Shamsudduha, M., [3]Taylor, R.G., [4]Zahid, A., [5]Ahmed, K.M., [6]Mukherjee, A., [7]Lapworth, D.J. and [8]Bense, V.

* Correspondence to william.burgess@ucl.ac.uk
[1] Department of Earth Sciences, University College London, London WC1E 6BT, UK
[2] Institute for Risk and Disaster Reduction, University College London, London WC1E 6BT, UK
[3] Department of Geography, University College London, London WC1E 6BT, UK
[4] Bangladesh Water Development Board, Dhaka, Bangladesh
[5] Department of Geology, Dhaka University, Dhaka 1000, Bangladesh
[6] Department of Geology and Geophysics, Indian Institute of Technology Kharagpur, West Bengal 721302, India
[7] British Geological Survey, Wallingford, Oxfordshire OX10 8BB, UK
[8] Department of Environmental Sciences, Wageningen University, The Netherlands

Supplementary Information

SI 1. Site locations and piezometer details

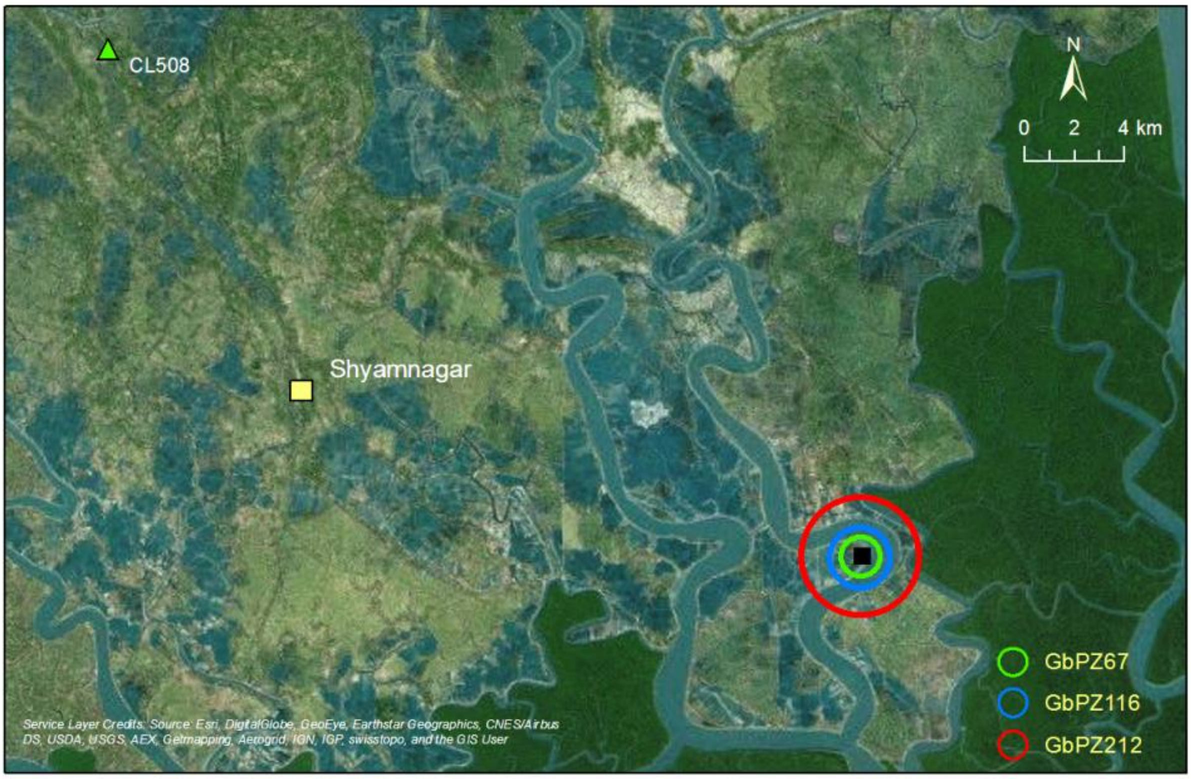

Figure SI 1a. Location of the Gabura multi-level piezometer site at latitude 22.27887, longitude 89.31125, showing its proximity to tidal channels and the approximate extent of the GbPZ67, GbPZ116 and GbPZ212 piezometer sensing areas. The BWDB rain gauging site CL508 is also indicated. The map was created using ArcGIS version 10.3.1 (https://www.arcgis.com/).

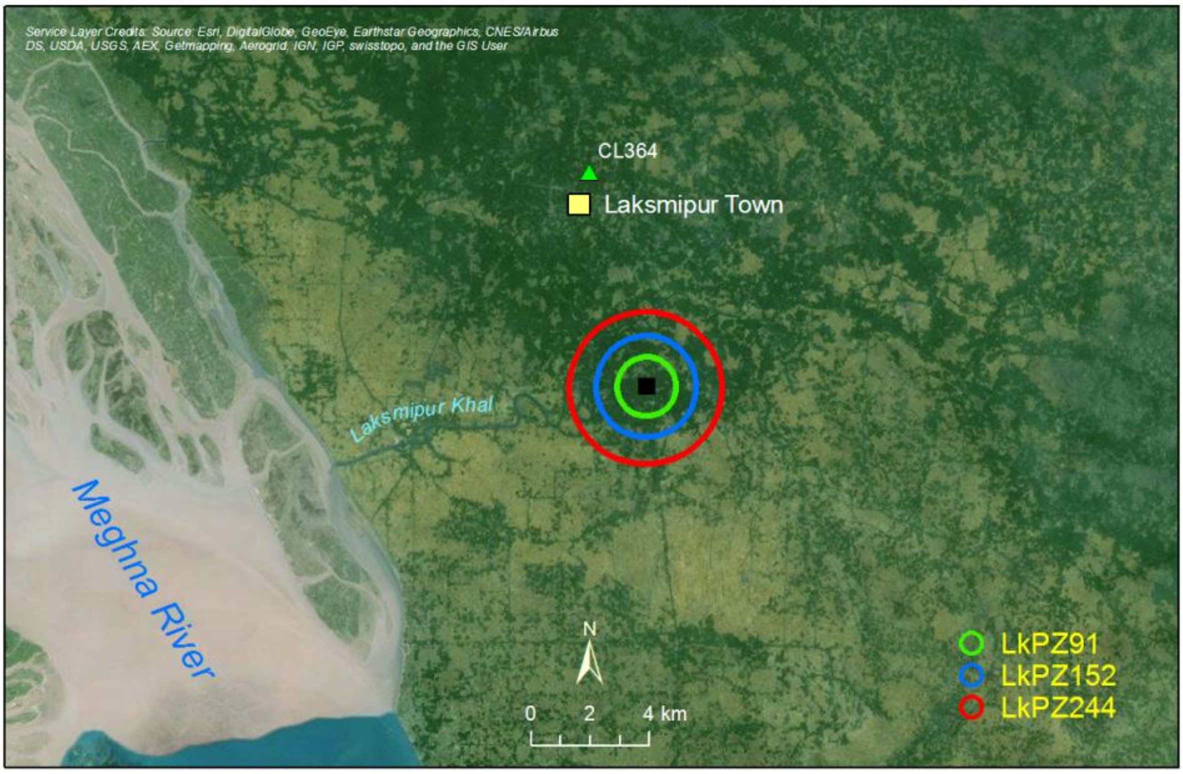

Figure SI 1b. Location of the Laksmipur multi-level piezometer site at latitude 22.88896, longitude 90.85750 showing its position relative to the Meghna River and Laksmipur town, and the approximate extent of the LkPZ91, LkPZ152 and LkPZ244 piezometer sensing areas. The BWDB rain gauging site CL364 is also indicated. The map was created using ArcGIS version 10.3.1 (https://www.arcgis.com/).

SI 2. GRACE seasonal TWS accumulation centred on Laksmipur, GBM floodplains

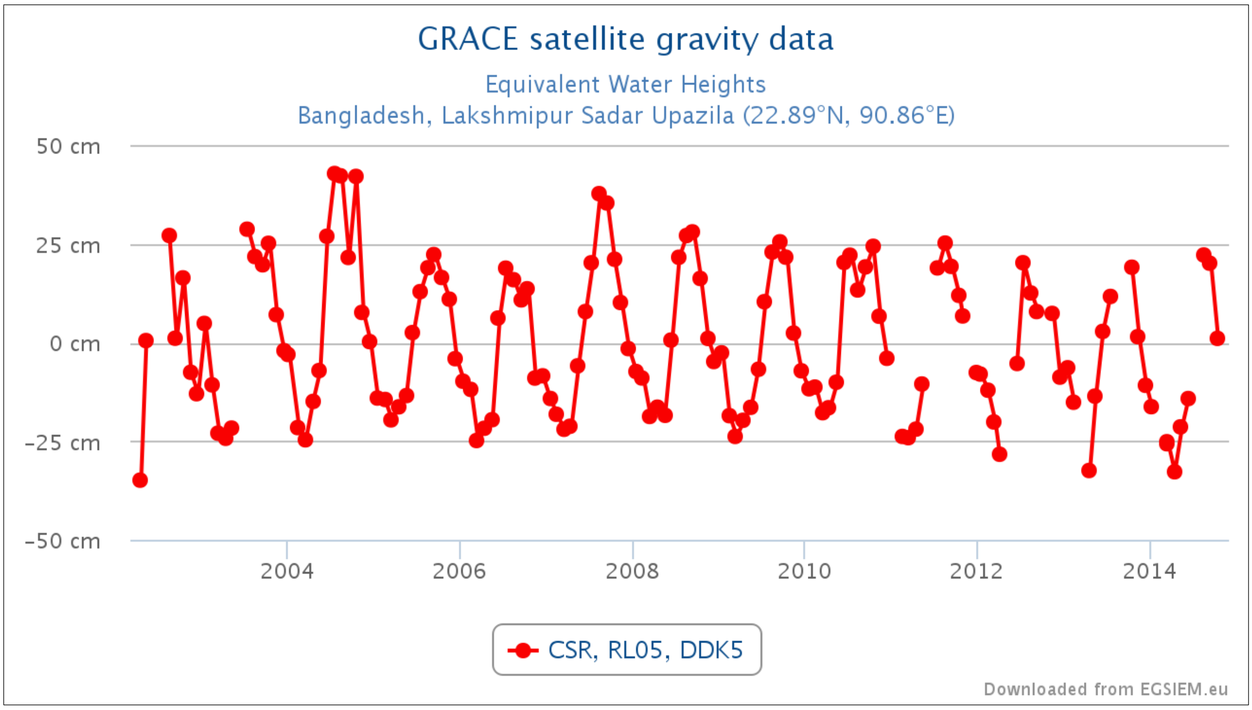

Figure SI 2. GRACE time-series data for ΔTWS from the European Gravity Service for Improved Emergency Management (EGSIEM; http://plot.egsiem.eu/), extracted for Laksmipur, 2003-2014. The TWS accumulation centred on Laksmipur over the 2013 monsoon season is 0.51 m; note that this is indicative, as GRACE data represent basin-scale variation of terrestrial water storage.

SI 3. Model simulation of the influence of poroelasticity, for a uniform aquifer representation and range of values of loading efficiency, *C*

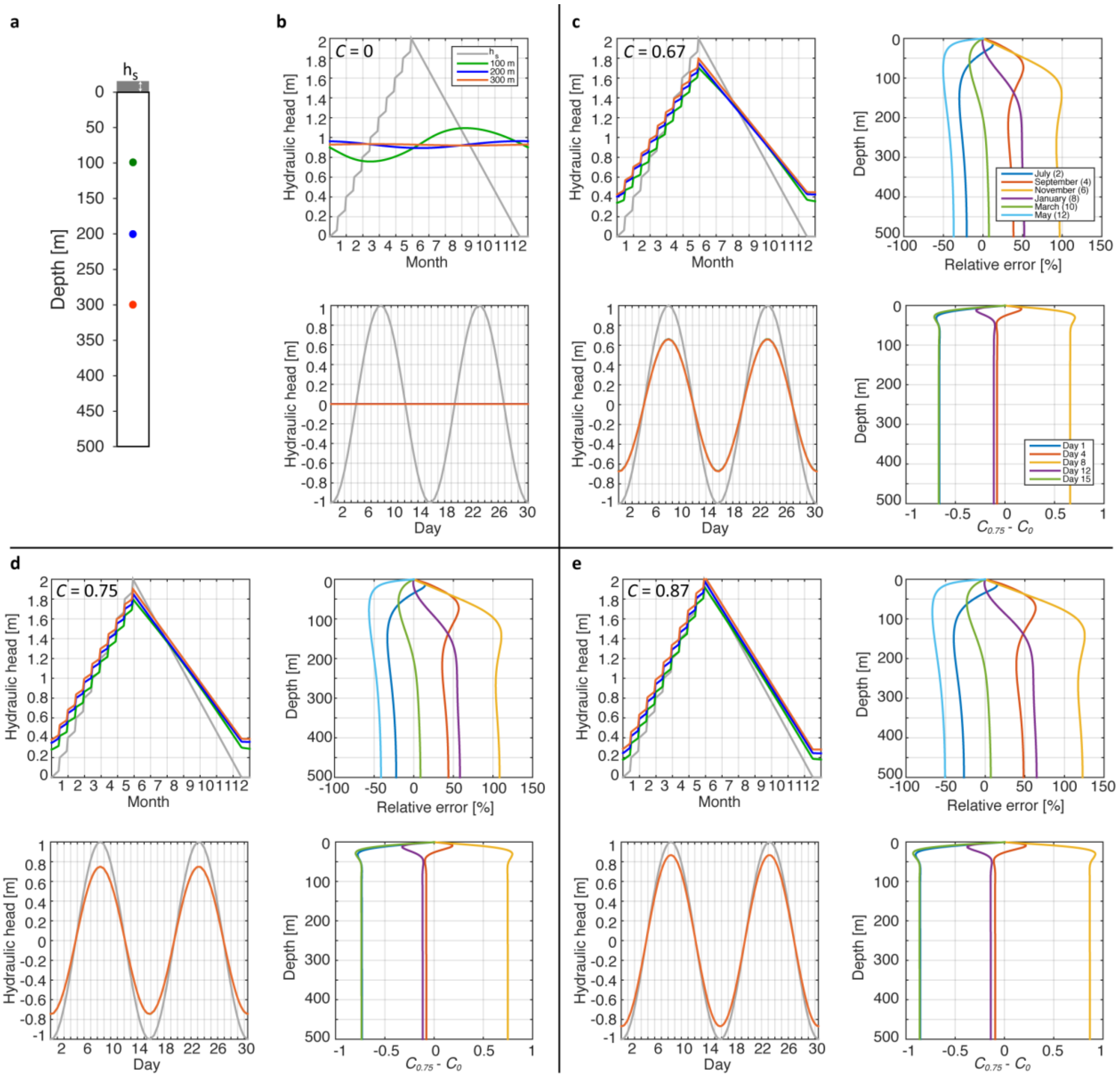

Figure SI 3. Model simulations showing the influences of poroelasticity for a uniform aquifer representation equivalent to the layered representation of Figure 5, with parameters K=4e-8 m/s, S_s=10^{-4} m^{-1}. (a) Model outline. A column of fresh water at the surface (h_s) represents surface water depth. Colour-coded dots at depths 100 m (green), 200 m (blue) and 300 m (red) show where hydraulic head is reported for a monsoonal scenario (top b, top left c, d, and e) and a tidal scenario (bottom b, bottom left c, d, and e) for different values of *C*. Values of loading efficiency, *C*, represent the range of uncertainty in this parameter, as described in the text. The relative differences between the monsoonal scenario simulations with finite values of *C* and with *C*=0 (top right c, d, and e) are expressed as relative error calculated *e.g.* as $\frac{C_{0.75}-C_0}{C_0}$; the absolute differences between the tidal scenario simulations with finite values of *C* and with *C*=0 are illustrated at bottom right of c, d, and e.
